# Ablating the aryl hydrocarbon receptor (AhR) in CD11c+ cells perturbs intestinal epithelium development and intestinal immunity

**DOI:** 10.1038/srep23820

**Published:** 2016-04-12

**Authors:** Song Hui Chng, Parag Kundu, Carmen Dominguez-Brauer, Wei Ling Teo, Kaname Kawajiri, Yoshiaki Fujii-Kuriyama, Tak Wah Mak, Sven Pettersson

**Affiliations:** 1 School of Biological Sciences, Nanyang Technological University, 60 Nanyang Drive, Singapore 637551; 2 Singapore Centre on Environmental Life Sciences Engineering (SCELSE), Nanyang Technological University, 60 Nanyang Drive, Singapore 637551; 3 Department of Microbiology, Tumor and Cell Biology (MTC), Karolinska Institutet, S-171 77, Sweden; 4 The Campbell Family Institute for Breast Cancer Research, Ontario Cancer Institute, University Health Network, Toronto, Ontario M5G 2C1, Canada; 5 National Cancer Centre of Singapore, Singapore 169610, Singapore; 6 Saitama Cancer Center Research Institute for Clinical Oncology, 818 Komuro, Inanmachi, Kitaadachi-gun, Saitama, 361-0806 Japan; 7 Tokyo Medical and Dental University, Medical Research Institute, 2-3-10 Kanda-Surugadai Chiyodaku, Tokyo 101-0062 Japan; 8 Lee Kong Chian School of Medicine, Nanyang Technological University, 11 Mandalay Road, Singapore 308232.

## Abstract

Diet and microbiome derived indole derivatives are known to activate the ligand induced transcription factor, the Aryl hydrocarbon Receptor (AhR). While the current understanding of AhR biology has confirmed its role in mucosal lymphocytes, its function in intestinal antigen presenting cells (APCs) is poorly understood. Here, we report that *Cre*-mediated deletion of AhR in CD11c-expressing cells in C57/BL6 mice is associated with altered intestinal epithelial morphogenesis *in vivo*. Moreover, when co-cultured with AhR-deficient DCs *ex vivo*, intestinal organoids showed reduced SRY (sex determining region Y)-box 9 and increased Mucin 2 expression, which correlates with reduced Paneth cells and increased goblet cell differentiation, similar to the data obtained *in vivo*. Further, characterization of intestinal APC subsets, devoid of AhR, revealed an expression pattern associated with aberrant intrinsic Wnt pathway regulation. At a functional level, the loss of AhR in APCs resulted in a dysfunctional epithelial barrier, associated with a more aggressive chemically induced colitis compared to wild type animals. Our results are consistent with a model whereby the AhR signalling pathway may participate in the regulation of innate immunity through intestinal epithelium development and mucosal immunity.

The gastrointestinal tract is constantly exposed to myriads of signals coming from our diet, the commensal bacteria and xenobiotics which all have an impact on host physiology. It is therefore interesting to think that a single transcription factor could bring together various information, be it endogenously or exogenously derived, to modulate gene expression in response to these signals. One potential candidate is the aryl hydrocarbon receptor (AhR), a ligand induced transcription factor. Besides industrial by-products^1^, planar indoles consisting of mainly tryptophan metabolites derived from the microbiome or from our diet (cabbage, broccoli and cauliflower) have been shown recently to activate the AhR^2^,^3^. Moreover, recent publications have placed AhR as a critical player in mucosal barrier defence, most notably for its role in IL-22 production in lymphocytes^4^. The current view supports the notion where the AhR functions as a sensor^5^,^6^, consistent with other members of the basic Helix-Loop-Helix/Per-Arnt-Sim (bHLH/PAS) family of transcription factors such as HIF-1α, which senses oxygen levels within a cell.

The mucosal immune system is tasked with maintaining the integrity of the epithelial barrier that provides both physical and biochemical restriction to unwanted luminal components from entering the lamina propria. Mucosal dendritic cells (DCs), armed with diverse signalling machineries are specialised to maintain intestinal homeostasis and are key to preserving a tolerant environment^7^,^8^. In addition, intestinal DCs and/or macrophages residing in the lamina propria are known to sample antigens found in the intestinal lumen via dendrites extending through inter-epithelial spaces^9^,^10^ or from goblet cells^11^ directly. Interestingly, DCs and macrophages have been reported to express the AhR protein, at levels similar to those found in lymphocytes^12^. Although the role of AhR in lymphocyte differentiation and function has been well described^6^, its function in intestinal antigen presenting cells (APCs) remains unclear.

The intestinal epithelial lining is highly dynamic and replenishes itself completely every three to five days^13^. Leu-rich repeat-containing G protein-coupled receptor 5 (Lgr5) –positive, crypt base columnar cells are stem cells that can undergo asymmetric division for self-renewal and to give rise to daughter cells that go on to repopulate the gut epithelium at regular intervals. This replacement program is constitutive, but sensitive to changes within the microenvironment. For example, restriction of calorie intake in mice was found to boost the size and activity of the stem cell pool via nutrient-sensing mechanisms within the stem cell niche^14^. The over-arching signalling mechanisms that regulate Lgr5+ stem cell biology and epithelium homeostasis rely mostly on two signalling pathways; Wnt signalling and Notch signalling^15^. While activation of both signalling cascades are necessary in maintaining the stem cell pool, the differentiation program of progenitors may reflect a disparity in preference, often in an opposing fashion^16^. In general, Notch activation pushes the precursors toward an absorptive phenotype while Wnt activation drives precursors toward a secretory cell lineage. Fate decisions in this scenario resemble a fine equilibrium that can be tipped to either side, where non-cell-autonomous signals coming from neighboring cells could also participate in. Consequently, cells located within the intestinal stem cell niche, which includes epithelial, stromal and hematopoietic lineages are key contributors to the combinatorial instructions regulating stem cell maintenance and differentiation. Hence, as Wnt signalling components are expressed by mucosal DCs^17^,^18^ and macrophages^18^, it is possible that these Wnt molecules may participate in the lineage decisions of intestinal precursors and/or stem cell maintenance. Moreover, the current literature provides evidences for the existence of cross-talk between the AhR and Wnt/β-catenin pathways in liver progenitors^19^, prostate cancer cells^20^ and colon cancer cells^21^. Thus, it is likely that AhR could also interact with Wnt/β -catenin pathways in the context of intestinal APCs. However, such a connection has not been made and whether the interplay between these two pathways within intestinal APC subsets could modulate the development of intestinal epithelium in the same microenvironment remains largely unexplored.

Here, we assessed whether intestinal DCs and macrophages could sense the external environment through AhR and thereby influence intestinal homeostasis. In CD11c-specific AhR knockout (KO) mice (11c^AhR−/−^), deleting AhR preferentially in CD11c-expressing intestinal mucosal DCs and certain macrophage subsets, we demonstrate a moderate but specific increase in small intestinal epithelial stem cell numbers and atypical differentiation of epithelial precursors *in vivo*. These changes are associated with increased susceptibility of the 11c^AhR−/−^ mice to chemically induced epithelial injury and colitis. Further, intestinal organoids exposed to AhR deficient DCs in co-cultures were found to express reduced levels of mature secretory cell type markers, which was dependent on the ratio of co-cultured DCs to isolated crypts *ex vivo*. Our data underscores the unique role of intestinal DCs and macrophages in maintaining the integrity of the intestinal epithelium via sensing of the external environment through AhR.

## Results

### 11c^AhR−/−^ mice are highly sensitive to DSS-induced colitis

**Figure 1:**
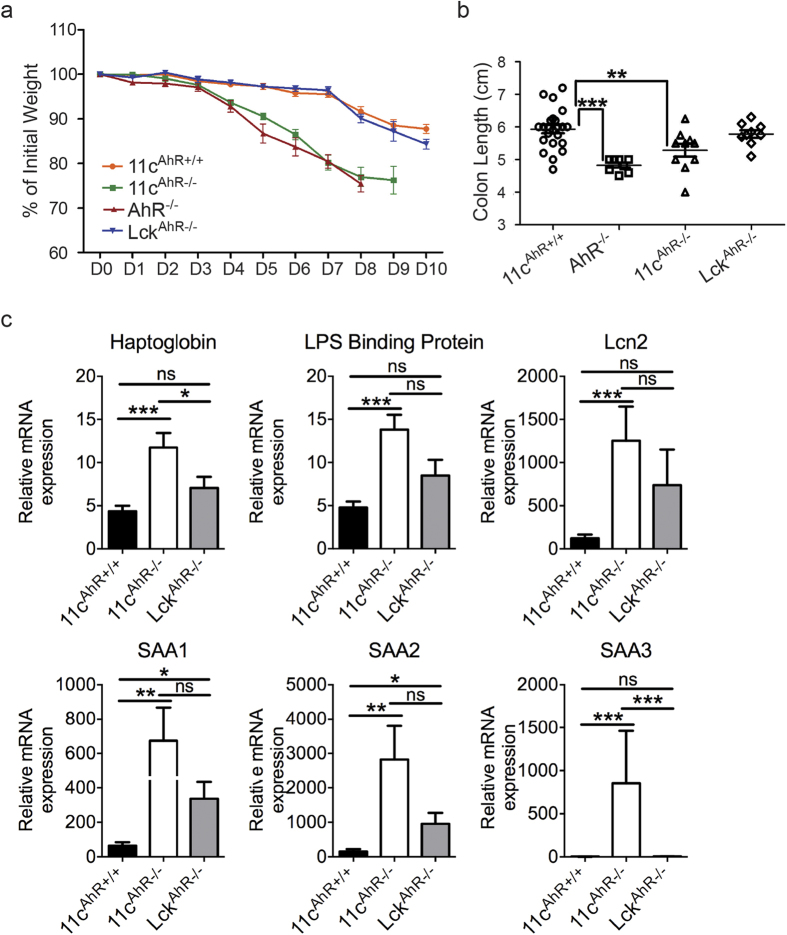
11c^AhR−/−^ mice are more susceptible to DSS-induced colitis. (**a**) The body weight of each mouse within various groups of mice were recorded daily and expressed as percentage of initial weight in grams over the course of the 2% DSS induced acute colitis. Data points are shown as mean ± SEM (n ≥ 8 per group). (**b**) Colon lengths of mice from various groups were measured in centimeters at end-point. Each symbol represents an individual mouse and horizontal lines show the mean ± SEM (n ≥ 8 per group), Student’s t test: **P < 0.01; ***P < 0.001. (**c**) Quantitative RT-PCR analysis of acute phase proteins genes expression in the liver post-DSS treatment. Bar graphs depict mean ± SEM (n ≥ 8 per group), Mann-Whitney test: *P < 0.05; **P < 0.01; ***P < 0.001; ns, not significant.

The involvement of intestinal APC subsets in modulating inflammatory responses as well as immune tolerance is undisputed. Here, we were interested in the function of AhR in these cells given that AhR KO mice were reported to be more susceptible to a plethora of intestinal challengers^22^,^23^. We ablated the environmental sensor and recently coined pathogen recognition receptor^5^, the AhR, specifically in both DCs and macrophages found in the intestinal lamina propria (LP). This was done by crossing a mouse where the *Ahr* gene is floxed in both alleles^24^ with another that expressed the *Cre* recombinase driven by the promoter of *Itgax* or CD11c to generate AhR-sufficient (11c^AhR+/+^) or AhR-deficient (11c^AhR−/−^) littermates for comparisons^25^,^26^,^27^. FACS sorted populations corresponding to DCs (R1 and R2) and macrophages (R3) showed efficient *Cre*-mediated deletion of *AhR* expression but not in CD11c+MHCII- cells within the lymphocyte gate, demonstrating specificity of the mouse line (Supplementary Figure S1). PCR reactions designed previously^24^ to detect the excised regions in targeted cells also showed clear excised bands only when *Cre* was present in FACS sorted DCs from the mesenteric lymph nodes (MLN) and the small intestinal LP (Supplementary Figure S1). Next, to challenge the 11c^AhR−/−^ mice, we supplied 2% dextran sodium sulphate (DSS) in the drinking water *ad libidum*, to induce acute inflammatory colitis. Interestingly, the 11c^AhR−/−^ mice showed considerably more weight loss and increased colon shortening compared to their controls, similar to the phenotype previously reported for AhR KO mice^22^ (Fig. 1a,b). Correlating with the heightened sensitivity to the acute inflammatory model, we also noted an elevation of acute phase protein genes expressions in the liver, suggesting systemic engagement of inflammatory processes in the 11c^AhR−/−^ mice compared to the control mice. Serum amyloid A1, A2 and A3 were increased by ~10, ~18 and ~233 fold respectively and haptoglobin, lipopolysaccharide-binding protein and lipocalin-2 expression were also massively increased in DSS treated 11c^AhR−/−^ mice compared with the controls (Fig. 1c). To assess whether these perturbed responses to DSS colitis were cell type specific, we generated T cell specific AhR KO mice (Lck^AhR−/−^) and exposed them to the 2% DSS challenge. No noticeable difference in the severity of DSS induced colitis between the Lck^AhR−/−^ mice and the 11c^AhR+/+^ controls was observed as shown in Fig. 1a–c and Supplementary Figure S2. No difference in the body weight of the 11c^AhR−/−^ animals versus littermate controls was detected (Supplementary Figure S2) at steady state. These data suggests that AhR in APCs plays a protective role in DSS-induced colitis.

### 11c^AhR−/−^ mice exhibited deregulated small intestinal epithelial cell development

**Figure 2:**
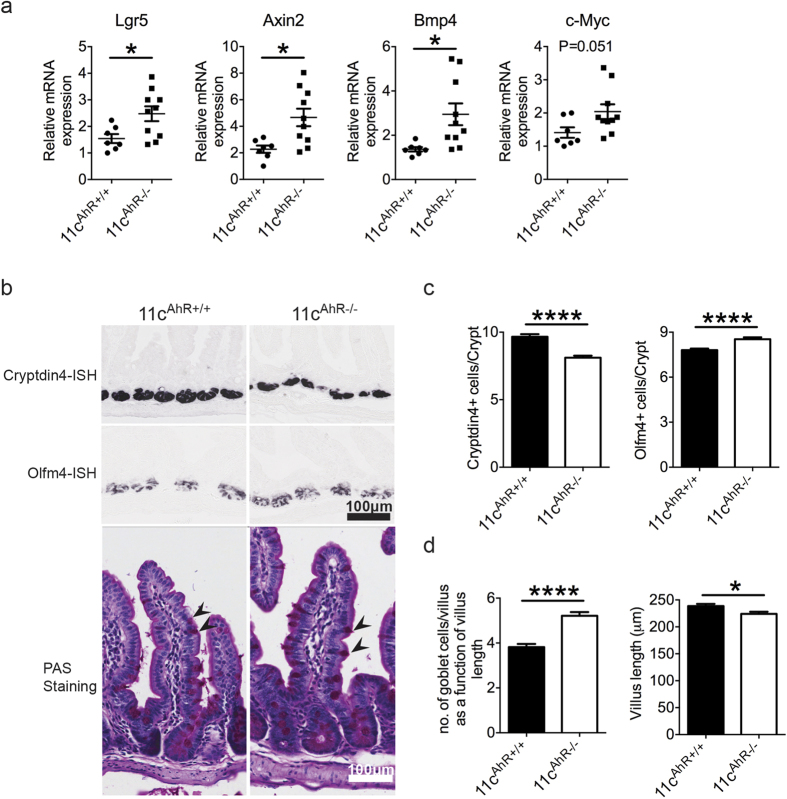
Altered intestinal epithelium morphogenesis in adult 11c^AhR−/−^ mice. (**a**) Quantitative RT-PCR analysis on Wnt-target genes expression from ileum epithelial scrapings. Data were pooled from 3 independent experiments and presented as mean ± SEM. Each symbol represents a single mouse. (**b**) *In situ* hybridization (ISH) and Periodic acid–Schiff (PAS) staining performed on paraffin-embedded sections of the ileum. Arrows point at stained goblet cells in the villus. (**c**) Quantification of intestinal stem cell and Paneth cell numbers. Graphs depict mean ± SEM of counted cells per crypt. More than 30 crypts were counted per animal (n = 4). (**d**) Quantification of goblet cells and villus length. Goblet cell numbers were counted and presented as a function of its respective villus length. Graphs show mean ± SEM (n = 3). Student’s t-test: *P < 0.05; ****P < 0.0001.

DSS has been widely used as an agent to disrupt intestinal barrier integrity, leading to inflammation. The increased susceptibility of 11c^AhR−/−^ mice to DSS induced epithelial damage suggests an altered intestinal epithelial landscape. Intestinal epithelial cells (IECs) take part in intestinal tolerance by secreting factors that crosstalk with the APCs^7^. This interaction is bi-directional where APC derived signals, in turn, influence intestinal epithelial cell biology and function. Together with the knowledge that Wnt pathway members are expressed by intestinal APC subsets^17^ and the increased sensitivity of 11c^AhR−/−^ mice to DSS challenge, we ensued to perform a pilot study on intestinal epithelial scrapings to investigate for perturbations in the Wnt pathway in IECs in the absence of AhR in intestinal APC subsets *in vivo*. Here, we noted a general increase in the expression of Wnt target genes such as axin inhibition protein-2 (Axin2), bone morphogenetic protein-4 (Bmp4), leucine-rich repeat-containing G-protein coupled receptor-5 (Lgr5) and cellular myelocytomatosis oncogene (c-Myc) via RT-PCR assays conducted on IEC scrapings isolated from the 11c^AhR−/−^ mice (Fig. 2a). Motivated by these findings that indicated aberrant Wnt signalling activities in IECs, we performed *in-situ* hybridisations using probes that specifically label intestinal stem cells (olfactomedin 4 or Olfm4) and Paneth cells (Cryptdin-4) in addition to carrying out PAS staining, which labelled mainly goblet cells (Fig. 2b and Supplementary Figure S3). We found a slight increase in both intestinal stem cell and goblet cell populations while Paneth cell numbers were reduced in the ileal epithelium of 11c^AhR−/−^ mice (Fig. 2c,d). Of note, the average villus length measured was shorter in the mutant mice compared to the control group (Fig. 2d).

### Attenuated *ex vivo* differentiation of secretory cell types in organoids exposed to AhR-deficient DCs

**Figure 3:**
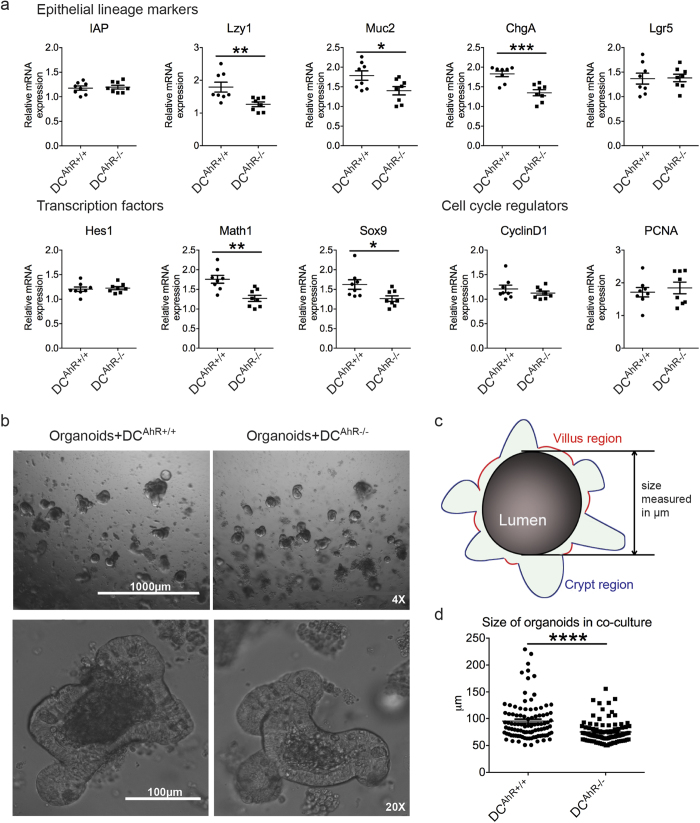
Differentiation of progenitors into secretory cell types are reduced in organoids co-cultured with AhR-deficient DCs. (**a**–**d**) CD11c+MHCII^hi^ DCs from the MLNs of 11c^AhR +/+^ or 11c^AhR−/−^ mice were pooled (n = 3) and co-cultured with isolated small intestinal crypts from AhR^fl/fl^ mice. Co-cultures were kept for five days. (**a**) Relative expression of epithelial lineage markers, transcription factors and proliferative markers were examined via quantitative RT-PCR. Each symbol represents a single biological replicate and data presented as mean ± SEM. Dataset shown was one out of two independent experiments performed with similar results. (**b**) Bright-field images of co-cultures are shown at different optical zooms. (**c**) Schematic showing how sizes of organoids were measured. (**d**) Size of organoids co-cultured presented as ±SEM from the two groups. Student’s t-test: *P < 0.05; **P < 0.01; ***P < 0.001; ****P < 0.0001.

The results we obtained raised an important question of whether the differences observed were a direct or indirect effect of AhR deficiency in intestinal APC subsets. To address this question, we first capitalized on a recently established protocol^28^ that facilitated the growth of isolated intestinal crypts, which contained stem cells that can indefinitely self-renew, proliferate and differentiate into all known epithelial lineages *in vitro*. Using the aforementioned protocol, we established co-culture systems involving isolated intestinal DCs and intestinal organoids in hopes to answer the above question. In this scenario, the ideal experiment was to isolate LP DC and macrophage subsets at numbers reasonable for co-culture experiments. However, due to technical reasons, we chose to work with CD11c+MHCII^high^ DCs isolated from the MLNs as they were more readily accessible and believed to have migrated from the LP^29^, making them the next best cells to test in our assay. Thus, we conducted the following experiments using MLN DCs harvested from either the 11c^AhR+/+^ or the 11c^AhR−/−^ littermates and co-cultured these cells with small intestinal crypts derived from AhR^fl/fl^ mice. The co-cultures were kept for a period of five days to allow epithelial precursors to differentiate into mature cell types over the course of the experiment. The start date of co-cultures was denoted as Day *in vitro* (DIV) 1 and co-cultures were stopped on DIV 5 accordingly. At DIV1, 3 and 5, we fixed some of the co-cultures and visualised for the presence of DCs embedded in the Matrigel via immunofluorescence staining. As shown in Supplementary Figure S4, DCs (red and arrowheads) counterstained with DAPI for nuclei were detected. Of note, images from DIV5 show DC with condensed nucleus, indicating a dead or unhealthy cell compared to cultures fixed at earlier time points, consistent with the short half-life of primary DCs. Following, we studied the markers for differentiated epithelial cell types and stem cells in organoids harvested at DIV 5. Markers used included intestinal alkaline phosphatase (IAP) for absorptive enterocytes, lysozyme 1 (Lzy1) for Paneth cells, mucin 2 (Muc2) for goblet cells, chromogranin A (ChgA) for enteroendocrine cells and lastly, Lgr5 for stem cells. Interestingly, while Lgr5 expression levels were similar comparing the two groups, we found that all markers for secretory cell types were significantly down regulated in organoids co-cultured with AhR-deficient DCs but not WT DCs (Fig. 3a). In agreement, the master transcription factor *Math1* required for the differentiation of all secretory cell-types was similarly down regulated, but not *Hes1* that supports enterocyte differentiation (Fig. 3a). Furthermore, SRY (sex determining region Y)-box 9 (Sox9), a transcription factor important for the differentiation of Paneth cells^15^ and a Wnt target gene^30^ was also found to be down-regulated, albeit only in one out of two independent experiments conducted (Fig. 3a). Expression levels of cell-cycle genes *CyclinD1* and *PCNA* did not reveal any statistically significant differences in proliferation (Fig. 3a), but the average size of the organoids cultured with AhR-deficient DCs at end point were found to be smaller (Fig. 3b–d). In addition, toward a more physiologically relevant level and for equitable comparisons, we went on to setup co-cultures with increased number of DCs to Crypt ratio at 5:1, plus a control group where organoids were grown without DCs. The results obtained were in a similar trend as to those reported differences at 1:1 ratio presented in Fig. 3a, as shown in Supplementary Figure S4. Accordingly, for Muc2 (goblet cell marker) and Sox9 (Paneth cell differentiation marker), one-way ANOVA with Dunnett follow up tests revealed statistically significant differences between the control and the DC^AhR−/−^ group but not with the wild-type DC^AhR+/+^ group (Supplementary Figure S4). Of note, relative expression of PCNA was also reduced in the DC^AhR−/−^ group compared to the DC^AhR+/+^ group in the same experiment (Supplementary Figure S4). Taken together, our data suggests a shortfall in Wnt signalling dependent differentiation and/or proliferation of IECs when AhR was absent in co-cultured DCs.

### AhR deficiency perturbs intestinal APCs homeostasis

**Figure 4:**
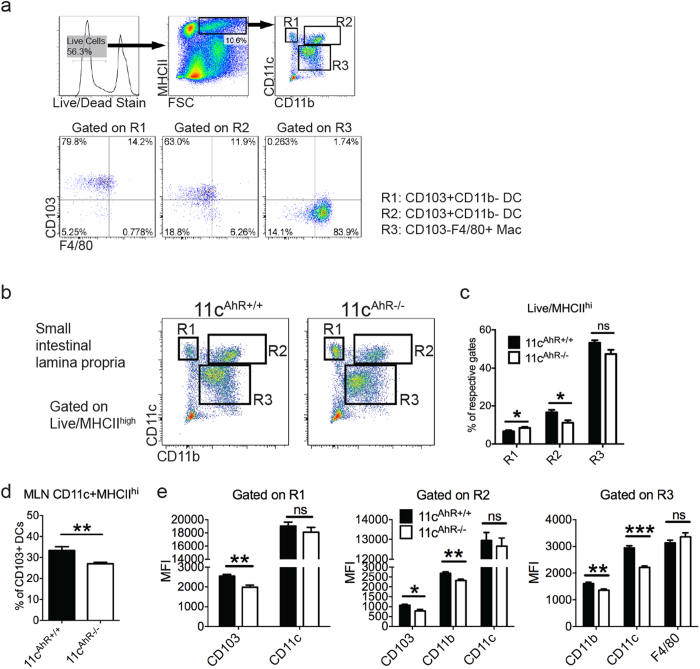
Classical cell-surface markers expressions in intestinal APCs were down regulated in the absence of AhR. (**a**) Live/MHCII^hi^ cells were gated and the three major subsets of APCs in the SI LP are presented as shown based on their expression of markers: CD11c, CD11b, CD103 and F4/80. (**b**–**c**) Quantification of the three major subsets of APCs as defined in (**b**), shown as percentages among total live/MHCII^hi^ cells. Results are mean ± SEM pooled from 5 independent experiments (n ≥ 10). (**d**) Quantification of MLN CD103+ DCs as mean percentages ± SEM is shown (n = 6). (**e**) Mean fluorescent intensity (MFI) representing expression levels of various cell surface markers of gated populations of all three groups are shown. Data are mean ± SEM pooled from 4–5 independent experiments (n ≥ 9). Mann-Whitney test: *P < 0.05; **P < 0.01; ***P < 0.001; ns, not significant.

**Figure 5:**
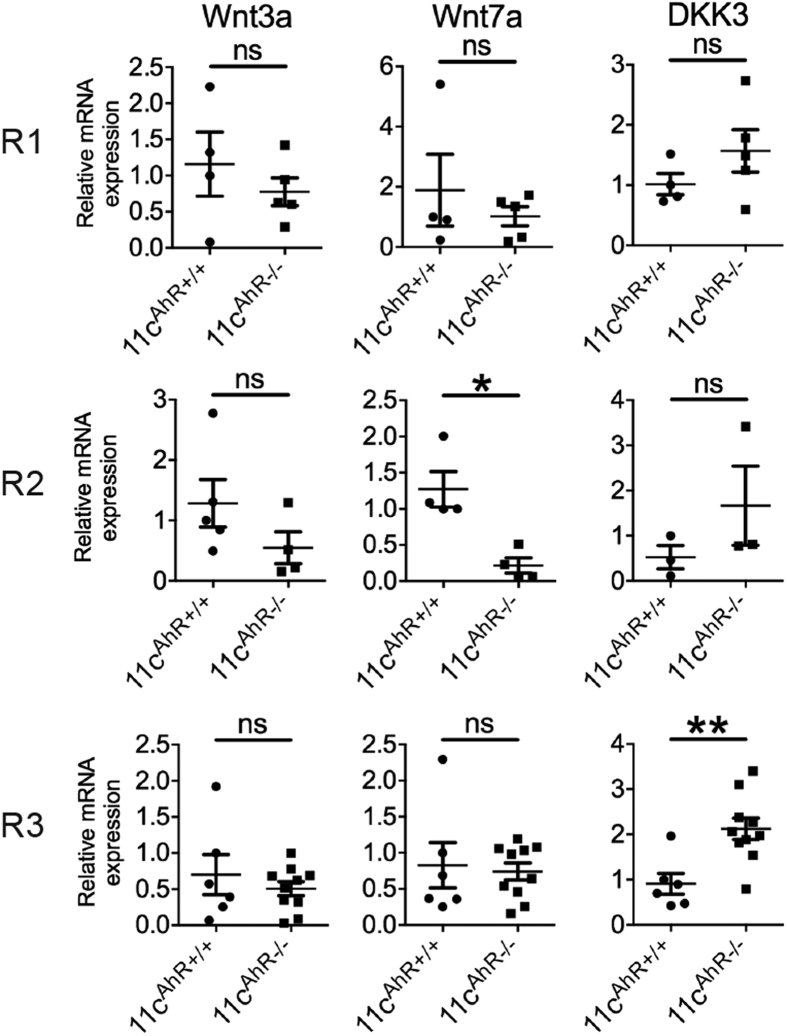
Intestinal LP APCs from 11c^AhR−/−^ mice exhibit aberrant expression of Wnt signalling constituents. Quantitative RT-PCR performed on cDNA generated from FACS sorted populations corresponding to R1, R2 and R3 APC subsets. Each symbol represents data from one single mouse. Data presented as ±SEM were pooled from 3–5 independent experiments. Mann-Whitney test: *P < 0.05; **P < 0.01; ns, not significant.

Prompted by these findings, we then focused on identifying aberrations, if any, in intrinsic Wnt signalling components of various intestinal APC subsets *ex vivo*. To begin, we first attempted to categorise the different subtypes of major APCs found within the LP where CD103 (integrin αE or *ITGAE*) positive APCs are the *bona fide* DCs while CD103 negative, F4/80 (*EMR1*) positive and CX_3_CR1 chemokine receptor high expressing cells are the macrophages in the LP at steady state^31^. Using three different gating strategies, we could differentiate the macrophages from the DC pool and further characterize the DCs into two distinct groups (Fig. 4a). Thus, the cells gated in R1, R2 and R3 represents CD103+CD11b− DCs, CD103+CD11b+DCs and CD103−F4/80+ macrophages respectively (Fig. 4a). From our FACS analysis data, we learned that in the absence of AhR, the expression of known cell-surface markers as mentioned above were largely irregular where all three subsets of APCs were affected (Fig. 4b,e). In addition, the percentage of R2 DCs was reduced; concomitant with a slight increase in the percentage of R1 DCs without significant changes in R3 gated macrophages detected (Fig. 4c). Since intestinal CD103+ LP DCs (R1 and R2 cells) are known to migrate into the MLNs at steady state, we analysed and found that the percentage of CD103+ DCs in the MLNs was decreased compared to control animals (Fig. 4d). Increased CD103 expression was shown previously via AhR activation in glioma cells^32^ and its down-regulation here in both CD103 expressing DC subsets (Fig. 4e) supports the deletion of functional AhR in the 11c^AhR−/−^ mice. Collectively, these results may reflect functional consequences of the loss of AhR in these cells at steady state. Subsequently, we FACS sorted the LP APCs into three distinct groups as described from the 11c^AhR−/−^ mice and compared their gene expression profile with those of their littermate controls. We performed these experiments using a micro-fluidics based RT-PCR system^33^ and a pre-selected list of genes (Supplementary Table 1), paying particular interest to those in the Wnt pathway. Interestingly, we found that the expression of Wnt7a was down regulated in CD103+CD11b+(R2) DCs but not in CD103+ CD11b− DCs (R1) isolated from the 11c^AhR−/−^ mice (Fig. 5). Conversely, the expression of a member of the dickkopf Wnt signalling pathway inhibitor (Dkk), Dkk3 was significantly up regulated in AhR-deficient intestinal macrophages (Fig. 5). Although significant differences in the expression of Wnt components were observed, we would caution that this is not a direct representation of the levels of secreted Wnt proteins.

### Immunophenotyping of 11c^AhR−/−^ mice did not reveal marked mucosal T cell immunity

**Figure 6:**
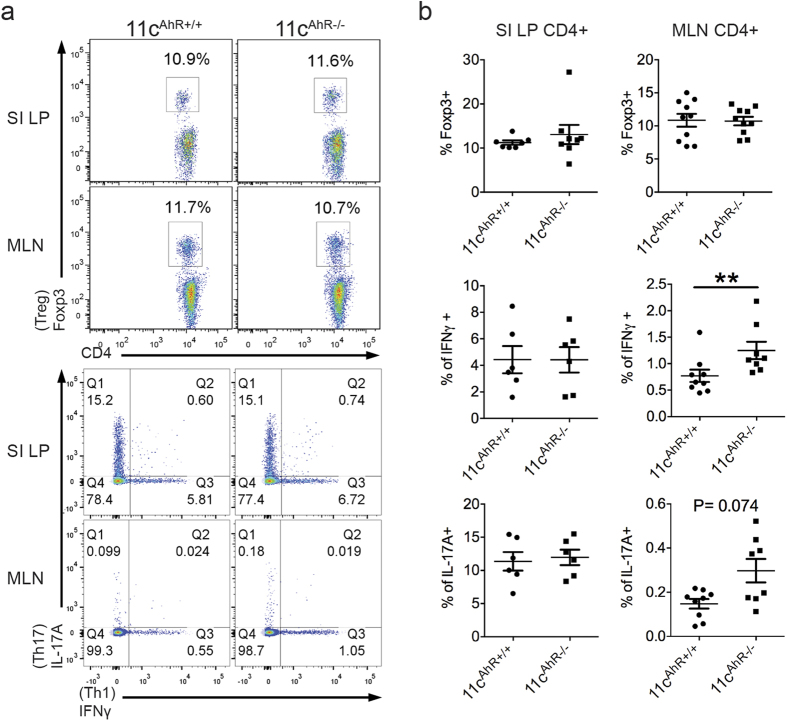
AhR-deficiency in intestinal APC subsets did not result in overt mucosal immunity. (**a**,**b**) Isolated lymphocytes from the small intestinal LP or the MLN re-stimulated for 4 hours *ex vivo*. (**a**) Representative FACS plots showing intracellular staining for Foxp3, IFNγ or IL-17 A in CD4+ T cells. (**b**) Graphs illustrate mean percentages within the CD4+ population ±SEM. Each symbol represents one mouse. Data were pooled from 3–4 independent experiments. Mann-Whitney test: *P < 0.05; **P > 0.01; ns, not significant.

Since Wnt/β-catenin signalling in DCs is crucial for intestinal tolerance^17^, our results directed us to study the activation status of these cells as well as known markers associated with tolerogenic DCs and/or macrophages. Based on our FACS analysis and expression datasets, we saw an up regulation of activation markers (CD86 and MHCII) in AhR-deficient intestinal macrophages and also a decrease in IL-10 expression in these cells (Supplementary Figure S5). For intestinal DCs, activation of these cells was less pronounced or no difference was observed when AhR was absent (Supplementary Figure S5). However, TGFβ1 and aldehyde dehydrogenase 1 family member A2 (ALDH1a2) were reduced in the R2 DCs (Supplementary Figure S5). The known high efficacy of intestinal CD103 + DCs in driving the development of Foxp3+ regulatory T cells (T_reg_) has been attributed to its ability to induce TGF-β and retinoic acid (RA) signalling (via the action of ALDH1a2) respectively, in responding naïve T cells^34^,^35^. Thus, our results here suggested a possible change in the LP T cell populations. Following, we did not find any significant differences in T_reg_, IL-17 and/or IFNγ producing CD4+ T cells in the small intestinal LP (Fig. 6a,b). We also did not observe any difference in the levels of fecal IgA, when comparing both groups of animals (Supplementary Figure S5). Nonetheless, in the MLNs, a two-fold increase in IFNγ producing CD4+ T cells was detected in the 11c^AhR−/−^ mice (Fig. 6a,b), indicative of a change in inflammatory status that was restricted to the lymphoid organ but not penetrating into the intestinal mucosal at steady state. Taken together, our results show that AhR signalling in intestinal APCs may be redundant for the maintenance of T_reg_ while the loss of AhR would be consistent with increased immune activation intrinsically at steady state.

## Discussion

In this study, we uncovered that the loss of an environmental sensor in intestinal sentinel cells under steady state conditions is associated with abnormal intestinal epithelial development and aberrant Wnt signalling in these APCs. This was coupled to heightened sensitivity to DSS, which is known to be toxic to intestinal epithelial cells, highlighting an intricate relationship between intestinal APCs and IECs during health and disease.

Cell types of different ontogeny (epithelial, stromal and hematopoietic lineages), found at barrier interfaces between the host and the external environment, are each armed with in part overlapping receptor mediated mechanisms to respond to the fast changing microenvironment^36^,^37^,^38^,^39^. These overlapping systems make mechanistic studies a true challenge as each cell-type often contribute, in its own way, in response to a given exogenous stimuli resulting in a combinatorial outcome. When we abrogated AhR in intestinal CD11c positive cells, the expression pattern of Wnt target genes increased along with an altered morphogenesis of the small intestinal epithelium of the 11c^AhR−/−^ mice as observed. Our data suggests that DCs and macrophages may play a role in modulating Wnt signalling of cells that are anatomically near or in close proximity such as the IECs. In support, a previous study has shown that intestinal DCs and macrophages are capable of participating in Wnt signalling, including the expression of Wnt ligands^17^, highlighting the possibility of a cross-talk via secreted Wnts between the immune cells and the epithelium at steady state. Following, a study by Koch and colleagues has shown that Dkk1 (another member of the dickkopf family of Wnt antagonists) was up regulated to the highest levels in DCs, relative to other hematopoietic lineages in the colon of DSS challenged mice^40^. Interestingly, Dkk1 KO mice were found to recover faster but develop ectopic crypt architectures during wound repair. Thus, the increased expression of Dkk3 by the R3 subset of APCs in our 11c^AhR−/−^ mice at steady state conditions may be of interest for future studies. Taken together, it seems that intestinal DCs and macrophages could dampen or perturb homeostatic Wnt signalling in neighbouring cells via intrinsic AhR activity.

Our *in vivo* data from the 11c^AhR−/−^ mice has shown a slight but statistically significant reduction in Paneth cell numbers per crypt and an increase in Olfm4+ intestinal stem cells. In some mouse models where Paneth cells are dysfunctional or absent, significant increase in Wnt target genes expression as well as increased Olfm4+ stem cells were observed^41^,^42^. Our results, presented here, mirrors what has been reported due to the reduction of Paneth cells in our 11c^AhR−/−^ mice. Furthermore, as differentiation of secretory subtypes is dependent on Wnt activation in general, the opposing effect on Paneth cells versus goblet cells in the small intestines of 11c^AhR−/−^ mice is noteworthy. Our co-culture experiments suggest that AhR in CD11c expressing cells could participate in modulating the specialisation of IECs via Math1, adding an additional layer of regulation. In the experiment where we increased the DC to crypt ratio, organoids co-cultured with AhR-deficient DCs had reduced expression of Sox9 but increased levels of Muc2 versus the control group. This result matches closely to the *in vivo* situation of reduced Paneth cells but increased number of goblet cells in the 11c^AhR−/−^ mice. Hence, differential Wnt activity levels and/or different Wnt ligands may account for the specialisation of different epithelial cell types observed. In support, the inhibition of β -catenin/Tcf4 activity has been demonstrated to promote goblet cell differentiation in colonic tumours^43^, highlighting the heterogeneity of Wnt activation on secretory cell type specification. Following, the reduced size of organoids may be a reflection of decreased proliferation as evidenced in the relative levels of PCNA expression when co-cultures were setup at a ratio of 5 DCs to 1 isolated crypt. However, the possibility of indirect effects of AhR ablation in APCs on IEC differentiation cannot be formally excluded. The intestinal stem cell niche comprises of not only the epithelial cells but also cells of stromal and hematopoietic origins^44^. Stromal cells such as myofibroblasts and fibroblasts are found encircling the crypt regions and in the lamina propria, play a pivotal role in mucosal immunity^45^ in addition to providing Wnt ligands within the stem cell niche for example^45^,^46^. The observed differences here, may be an indirect effect of AhR signalling in DCs and macrophages, where changes in cytokines and/or growth factors such as TGFβ1^47^,^48^,^49^ will have yet unknown consequence on surrounding sub-epithelial myofibroblasts thereby impacting the intestinal stem cell pool. Importantly, the exact mechanisms of action of specific Wnt ligands and Wnt antagonists on stem cell biology and/or differentiation are still debatable. Recent work has demonstrated that non-epithelial and non-stromal derived Wnts are sufficient in maintaining intestinal villus-crypt structures and homeostasis *in vivo*^50^. In other words, supplementary Wnt sources from the microenvironment such as those from LP DCs, macrophages and lymphocytes may further complicate the interpretation of Wnt-signalling and intestinal biology among others.

Both Paneth cells and goblet cells participate in controlling the resident microbes via secretion of anti-microbial peptides and mucins respectively. A change in the composition of these cells suggests a skewing of microbial populations in the 11c^AhR−/−^ mice, an observation demonstrated in an earlier study without any detectable changes to barrier integrity, albeit in the whole AhR KO animals^22^. Interestingly, goblet cells have been described to be capable of transferring antigens to CD103+ DCs for subsequent antigen presentation^11^, critical for adaptive immune responses in the gut against potential microbial invasion. Consequently, favouring goblet cell differentiation over Paneth cells, mediated by AhR-deficient APCs, raises the possibility that AhR could participate in immune regulation by increasing or decreasing antigen presentation depending on the availability of AhR ligands. Intestinal CD103+ CD11b+ (R2) DCs are known to be the major population among the CD103+ DCs in the small intestines^51^ and responds to luminal microbes by migrating into the epithelium for antigen sampling^9^. Recently, R2 DCs and CD103−CX3CR1+ (R3) macrophages have been reported to act cooperatively via gap junctions, important for tolerance induction via R2 DCs in the MLNs^52^. In parallel, R3 cells were the first to be identified to extend dendrites into the lumen to sample antigens^10^, thereby mediating the antigen transfer from macrophages to the DCs^52^. Corroboratively, our data that suggests aberrant Wnt signalling in both R2- and R3- but not in R1-gated AhR-deficient APCs highlights the unique environmental sensing functions of different SI LP APC subsets. A loss of function of AhR in sentinel cells could therefore predispose individuals to environmentally linked diseases where the intestinal epithelium plays a critical role. Nonetheless, future work aimed at understanding the effects of AhR activation on the secretion of Wnt signalling agonists and/or antagonists in various intestinal APC subsets is highly warranted.

In recent years, the AhR has been closely studied for its role in immunological responses, where intrinsic expression of AhR in various immune cells have been linked to allergy^53^, autoimmunity^54^, immune-invasion of tumorigenic cells^32^, mucosal immunity^4^ and immune disease tolerance^55^. However, as AhR ligands are structurally very diverse and together with evidences supporting ligand specific gene regulation as being cell type and context dependent^56^, the field has been plagued by the promiscuity of the AhR. Thus, we used a loss of function approach specifically in CD11c-expressing cells in hopes to alleviate confounding effects due to the use of different agonists to study the role of AhR in these cells. In this study, we observed deregulated cell-surface receptors expression including elevated co-stimulatory molecules via FACS analysis of different intestinal LP APC subsets when AhR was absent. Previous studies using different AhR ligands or DCs derived from various tissue origins have reported varying results^49^,^57^,^58^. Yet the results are consistent as AhR activity was shown to regulate the differentiation and/or maturation and hence function of these APCs. Following, ligand induction of AhR, has been shown to induce tolerogenic CD103+ DCs, preferentially driving T_reg_ responses in a RA dependant manner to which ameliorate disease progression in a mouse model for multiple sclerosis^54^. The immune-regulatory effect of AhR activation in DCs was further demonstrated when immuno-suppressive IL-10 cytokine secretion upon LPS stimulation in AhR-deficient bone marrow derived DCs was reduced compared to wild-type controls^47^. Also, it was reported that in most cases, the immune-dampening effects of ligand induced AhR signalling in inflammatory conditions was attenuated when AhR is specifically ablated in the DCs^25^,^26^. Interestingly, specific ablation of AhR in DCs and some subsets of macrophages did not result in overt inflammation in a skin inflammation model, suggesting tissue restricted effects of AhR^27^. In parallel, we found that DCs in the small intestinal LP of the 11c^AhR−/−^ mice expressed lower levels of TGFβ1 and ALDH1a2 while macrophages expressed less IL-10 respectively in the absence of AhR at steady state. Surprisingly, we found that T_reg_ populations or fecal IgA levels from 11c^AhR−/−^ mice when compared to their littermate controls were not significantly different. We speculate that compensatory mechanisms might be at play to explain our findings for example, intestinal epithelial cells could contribute via the provision of proliferation-inducing ligand (APRIL) and B cell-activating factor (BAFF) upon Toll-like receptor activation to promote IgA secretion from plasma cells^44^ while stromal cells may also contribute to the generation of T_reg_ cells via various mechanisms^45^. In agreement, intestinal T_reg_ populations in AhR KO mice were at similar levels as wild type controls^22^, highlighting the role of other cell types in maintaining an immuno-tolerant environment at steady state. Moving on, while we did not find any differences in inflammatory CD4+ T cells in the LP of 11c^AhR−/−^ mice, we found a two-fold increase in CD4+ IFNγ+ T cells in the MLNs. Our mice did not get sick or display signs of weight loss compared to controls, suggesting that the tilt towards Th1 immunity is environmentally-dependent, as illustrated by the development of spontaneous colitis in AhR KO mice bred in certain animal facility^59^ but not others^60^,^61^. Collectively, it appears that the AhR plays a crucial role in APC function by shaping their preference towards immunity or tolerance in response to environmental cues.

The administration of AhR ligands have been shown to ameliorate colitis severity in various experimental models^62^,^63^,^64^. A protective role of AhR signalling in controlling chronic intestinal inflammation was further demonstrated by complementary studies highlighting the increased susceptibility of AhR KO mice to chemically induced colitis^22^,^65^. Interestingly, the increase in acute phase response genes during DSS colitis as well as the increase in goblet cell numbers in the 11c^AhR−/−^ mice detected resembled phenotypes reported in patients with Crohn’s Disease (CD) but not Ulcerative Colitis (UC)^66^,^67^. Also, significant reduction of AhR protein in the inflamed areas of the bowel in patients suffering from CD but not UC has been observed^68^. Taken together, the 11c^AhR−/−^ animals may be an interesting model to examine the pathogenesis of distinct categories of inflammatory bowel diseases.

The notion of retrospective interaction from the DCs to the intestinal epithelial cells has only recently garnered more attention, shown in a model of intestinal inflammation^69^. The current body of research has focused more on signals originating from the intestinal epithelial cells^44^ or mesenchyme cells^45^, acting on the immune system but seldom the reverse. This could be due to the classical view that immune subsets are mostly involved in inflammatory pathways via the secretion of cytokines and chemokines and not participate actively in the context of homeostatic regulation. Our findings provides an interesting aspect on how cell types of distinct developmental origins could sense and provide ‘common’ signals (possibly via Wnt signalling) to find the right balance between stem cell maintenance, proliferation and/or differentiation in IECs, in response to the changing external environment.

In summary, our data stressed the critical involvement of AhR signalling in the homeostasis of intestinal APC subsets, consequently modulating intestinal epithelium morphogenesis and mucosal immunity. The abundance of AhR ligands in the intestinal tract and the fact that the AhR has been evolutionarily conserved from nematodes to humans imply an essential role for AhR to facilitate APC adaptation to maintain intestinal homeostasis in mammals.

## Materials and Methods

### Mice

*Itgax*-*Cre* and *Lck*-*Cre* mice purchased from The Jackson Laboratory were crossed with AhR^fl/fl^ mice on a B6 background to generate dendritic cell specific (11c^AhR−/−^) or T cell specific (Lck^AhR−/−^) AhR KO mice. Mice aged 8–14 weeks were used for experiments. All mice were bred and housed at the SingHealth Experimental Medicine Centre, Singapore. This study was conducted in accordance with institutional guidelines at the Singhealth Experimental Medicine Centre, Singapore. All animal protocols performed were approved by the Singhealth institutional animal care and use committee.

### Dextran sodium sulphate induced colitis

Mice aged 8–10 weeks were given 2% DSS (TdB consultancy AB) in their drinking water for 10 days, which were replaced with fresh preparations every third day. The body weights of mice were monitored daily from day 0 where DSS was given on Day 1. Colon lengths were measured in centimetre scale, livers were harvested, snap-frozen and stored at −80 °C at end point.

### Intestinal epithelium scrapings and real-time PCR

Intestines were excised, opened longitudinally and fecal contents washed off using cold PBS. The small intestine was divided into 5 equal lengths and the most distal section was denoted as the ileum. Scrapings were performed using sterile blades on the surface of the intestines lightly, leaving the muscular layers intact. Tissues collected were snap-frozen in liquid nitrogen to minimize RNA degradation. Thereafter, total RNA was isolated from frozen tissues using RNeasy Mini Kit (Qiagen). 2 ug of total RNA was reverse transcribed to cDNA using the iScript cDNA synthesis kit (Biorad). Thermal cycling condition was performed as follow: 25 °C for 5 mins, 42 °C for 30 minutes and reaction terminated at 85 °C for 5 minutes. cDNA was pre-diluted with dH_2_O to a concentration of 10 ng/ul where 1 ul was used per RT reaction. Detection method used was based on Fast SYBR Green Master Mix reagent (Applied Biosystems) chemistry and the ABI 7500 fast real-time PCR system (Applied Biosystems). Relative gene expression analyses were done using the ∆∆Ct method with normalization to *Hprt*. The sequences of primers used are presented in Table S1.

### *In situ* hybridization

The *in situ* probes targeting *Olfm4* and cryptdin4 used in this study were previously described^14^. To ensure probe specificity, both sense and antisense probes were generated by *in vitro* transcription using DIG RNA labeling mix (Roche) according to previously published detailed methods^70^.

### Goblet cell counting

Periodic acid-Schiff staining on paraffin-embedded intestinal sections was done and slides were subsequently scanned and visualised using the Aperio ImageScope software. Stained goblet cells were counted per villus and the respective villus length was measured. At least 15 villi were examined per mouse in blind fashion.

### Isolation of intestinal lamina propria leukocytes

Peyer’s patches were first removed from the small intestines and thereafter, the intestines were opened longitudinally, cut finely and washed vigorously with ice-cold HBSS. The epithelial layer was then disrupted via incubation for 20 minutes in HBSS with 5% FBS, 2.0 mM EDTA and 1 mM DTT at 37 °C with agitation, followed by 20 minutes incubation in HBSS with 5% FBS and 2.0 mM EDTA with agitation. Following each incubation step, tissues were vortexed briefly and the supernatant was discarded. Following, tissues were washed and then minced and incubated at 37 °C for 1.5 hours in complete IMDM (Invitrogen) with 10% FBS, P/S and collagenase D at 1.5 mg/ml (Roche) with shaking. Digested tissues were homogenised and supernatants were first passed through a 100- and then 40-μm cell strainer and pelleted by centrifugation. Leukocytes were then enriched using a discontinuous Percoll (GE healthcare) gradient. After centrifugation at 2,800 rpm for 20 minutes at room temperature with zero deceleration, cells were collected at the 40/70 interface. Collected cells were washed twice and re-suspended in cold PBS with 2% FBS.

### Flow cytometry, staining and PCR

Isolated intestinal LP cells were routinely treated with anti-Fcγ receptor antibody for 5 mins on ice before staining with antibodies or corresponding isotype controls from eBioscience for 30 mins on ice unless otherwise specified: FITC-anti-CD11b (M1/70; BioLegend), PE-anti-CD11c (N418), PerCP-Cy5.5-anti-MHC II (I-A/I-E, M5/114.15.2; BioLegend), APC-anti-CD103 (2E7), APC-anti-CD80 (16-10A1), APC-anti-CD45 (30-F11), PE-Cy7-anti-F4/80 (BM8), APC-Cy7-anti-CD86 (GL-1; BioLegend), V500-anti-MHC II (I-A/I-E, M5/114.15.2; BD Biosciences). Live cells were determined using the LIVE/DEAD Fixable Dead Cell Violet Stain Kit (Invitrogen) or DAPI staining. All flow cytometry assays were performed on FACS Canto II or Aria II for sorting (BD Biosciences) and analysed with FlowJo software (Tree Star). Genomic DNA/RNA from FACS sorted DC populations were isolated using AllPrep DNA/RNA Mini Kit (Qiagen) and excision of floxed AhR exon 2 were confirmed by PCR following previous publication^24^.

### *Ex vivo* cultures and intracellular staining of CD4+ cells

Intracellular Foxp3 staining was performed using the Foxp3 fixation/permeabilization staining kit (eBioscience). For *ex vivo* cultures, cells from the MLN or the LP were stimulated with cell stimulation cocktail (eBioscience) containing PMA, ionomycin, brefeldin A and monensin for 4 hours at 37 °C with 5% CO_2_. Following, surface staining with FITC-anti-CD4 (RM4-5) and then intracellular staining with PE-anti-IL-17A (ebio17B7) and APC-anti-IFNγ (XMG1.2) was done using IC fixation buffer and permeabilization buffer (eBioscience).

### Isolation of small intestinal crypts and co-culturing with mesenteric lymph node dendritic cells

Intestinal crypts were harvested according to previously published protocol with slight modifications^46^. Briefly, whole small intestines were opened longitudinally, washed and cut into fine pieces. Then, tissues were left in cold PBS with EDTA (2 mM), followed by washing with cold PBS with agitation for repeated rounds to remove the villus while enriching for crypts in the supernatant. Collected supernatants containing mostly crypts were spun down at 200 g for 2 mins at 4 °C with soft brake on to facilitate removal of single cells during washes. While crypts were being prepared, mesenteric lymph nodes were collected, dissected and digested for 30 mins with Collagenase D (Roche) at 1.0 mg/mL. Dendritic cells were then enriched using the AutoMACS separator (Miltenyi Biotec) and anti-mouse CD11c beads. Cells were then stained and CD11c+ MHCI^hi^ cells were FACS sorted using FACS Aria II. Isolated crypts and DCs were mixed together at 1:1 or 5:1 DC to isolated crypts in 50 μL of matrigel (Corning Incorporated) droplet seeded onto each well of a 48-well plate. Co-cultures were maintained for five days where seeding day was considered day one in Advanced DMEM/F12 (Life Technologies) media with HEPES buffer plus supplements (N2 and B27), glutaMAX (Life Technologies), Y-27632, N-acetyl-L-cysteine (Sigma Aldrich), Penicillin/Streptomycin and recombinant proteins EGF, Noggin (Peprotech) and R-spondin-1 (R&D Systems). Subsequently, co-cultured organoids were treated with Trizol reagent (Life Technologies) for isolation of total RNA and stored at −80 °C for use later.

### Immunofluorescence staining

Co-cultures were fixed with 2% PFA for 10 minutes and washed with PBS before staining with anti-mouse-CD11c-PE and DAPI (1 μg/mL) in FACS staining buffer (2% FBS in PBS) for an hour at room temperature. Care had to be taken in between washes, as Matrigel (Corning Incorporated) tend to depolymerise upon fixation. Images were taken from fixed co-cultures at Days *in vitro* (DIV) 1, 3 and 5 using the Nikon Eclipse Ti inverted fluorescence microscope at 40X objective.

### FACS sorting for gene expression analysis

Intestinal LP leukocyte preparations were first cleared further of dead cells using the dead cell removal kit (Miltenyi Biotec). Following, CD11c + LP cells were magnetically enriched using anti-CD11c beads (Miltenyi Biotec) and cells were sorted using the FACS Aria II (BD Biosciences) to a purity of >98%. Due to limited cell numbers per mouse, 500 single cells/events per APC subset from a single mouse were sorted into individual 0.2 ml tubes for direct cell-lysis and subsequent reverse-transcription to generate cDNA for downstream RT-PCR assays as described previously^71^. In brief, cell lysis was done on ice, in 0.2 mL tubes containing 10U RNaseOUT enzyme (Invitrogen), 0.15% (v/v) Tween-20 (Biorad) and 0.2 mM DTT at a final volume of 12 ul with addition of nuclease-free water. For direct reverse transcription, 5X VILO Reaction Mix and 10X SuperScript Enzyme Mix (Invitrogen) were added to the cell-lysis tube to a final reaction volume of 20 ul. Thermal cycling conditions performed were as follow: 25 °C for 10 minutes, followed by 42 °C for 90 minutes and reaction terminated at 85 °C for 5 minutes. cDNA derived were subsequently used for microfluidics-based RT-PCR assay platform (Fluidigm). Specific target amplification (STA) was done prior to RT-PCR reactions. Resulting cDNA, 2X TaqMan PreAmp Master Mix (Applied Biosystems), 500 nM (10X) of pooled primers mixtures were topped up to a final volume of 5 ul with nuclease-free water followed by thermal cycling conditions as follow: 95 °C for 10 minutes, 14 cycles of 95 °C for 15 seconds, 60 °C for 4 minutes and finally hold at 4 °C. Unincorporated primers from the STA reaction were digested via exonuclease I (New England Biolabs) treatment and the resulting samples were diluted 5-fold for use in RT-PCR assays using the 48.48 Dynamic Array IFC on BioMark HD system (Fluidigm). The ∆∆Ct values were computed using the Real Time PCR analysis software (Fluidigm) with *Polr2α* as endogenous control. In the event where, for example, the R1 subset of DCs of a particular mouse did not reach the required 500 cells, the data point was excluded from analysis.

### Fecal IgA detection by ELISA

1–2 fecal pellets were collected in 2 mL tubes per mouse and weighed. Thereafter, 1X PBS containing complete protease inhibitor cocktail (Roche) were added to feces to achieve a final concentration of 100 mg/ml. Complete suspension of fecal pellets were ensured by grinding using pestles plus vortexing. Suspensions were allowed to stand on ice for an hour. Next, samples were centrifuged at 15,000 rpm for 15 minutes and supernatants were collected. Prior to loading, supernatants were pre-diluted 10 times. IgA concentrations were then determined using Mouse IgA ELISA Ready-SET-Go! Kit (eBioscience).

### Statistics

Student t tests (two-tailed), Mann-Whitney tests (two-tailed) and one-way ANOVA with Dunnett follow up tests were conducted using the Prism 6 (GraphPad) software.

## Additional Information

**How to cite this article**: Chng, S. H. *et al*. Ablating the aryl hydrocarbon receptor (AhR) in CD11c+ cells perturbs intestinal epithelium development and intestinal immunity. *Sci. Rep*. **6**, 23820; doi: 10.1038/srep23820 (2016).

## Supplementary Material

Supplementary Information
