# Supplementary material for: Ablating the aryl hydrocarbon receptor (AhR) in CD11c+ cells perturbs intestinal epithelium development and intestinal immunity
Source: Sci Rep. 2016 Apr 12;6:23820. doi: 10.1038/srep23820 (PMC4828637; doi:10.1038/srep23820)
Supplement: Supplementary Information [file srep23820-s1.pdf]

SUPPLEMENTARY INFORMATION

Title: Ablating the aryl hydrocarbon receptor (AhR) in CD11c+ cells perturbs intestinal epithelium development and intestinal immunity.

Authors' list: Song Hui Chng, Parag Kundu, Carmen Dominguez-Brauer, Teo Wei Ling, Kaname Kawajiri, Yoshiaki Fujii-Kuriyama, Tak Wah Mak, Sven Pettersson

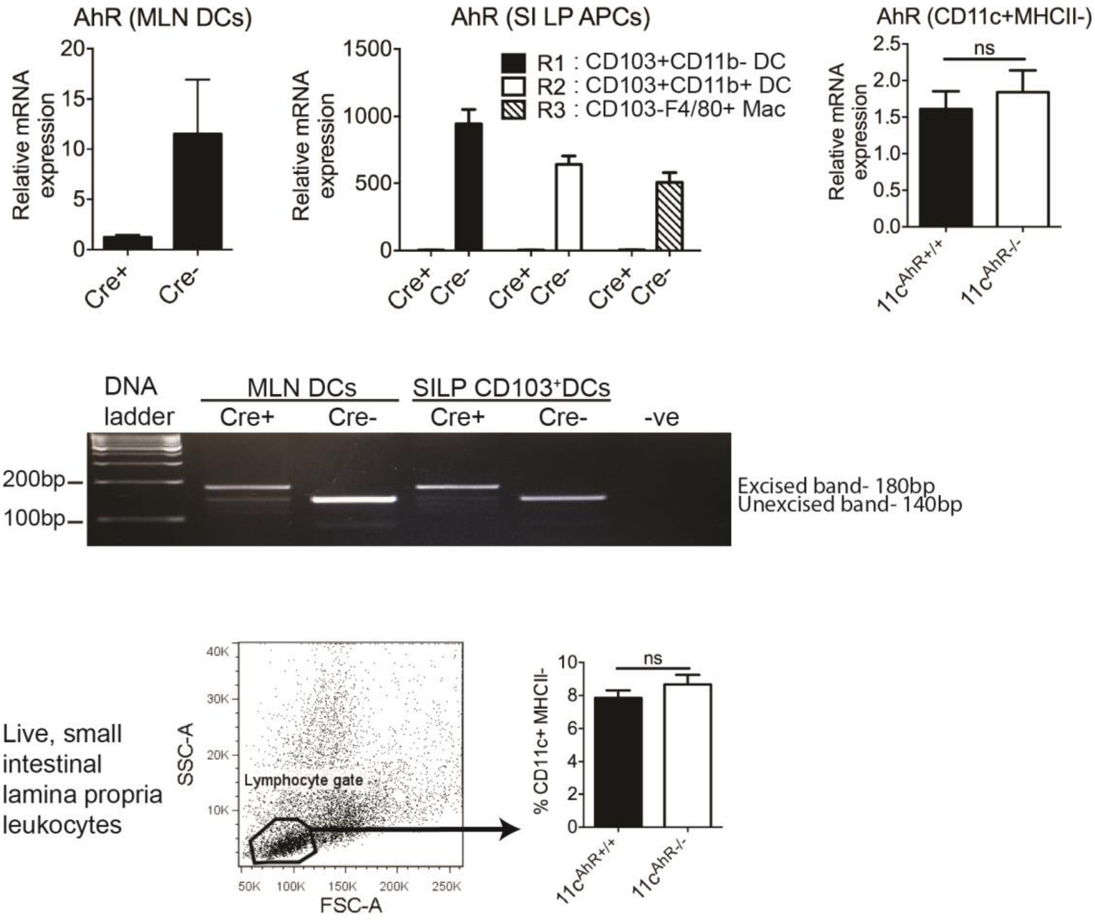

Supplementary Figure S1. Specificity and efficacy of *Cre*-mediated AhR deletion in 11c$^{AhR-/-}$ mice. Relative *Ahr* expression by quantitative RT-PCR assays on MLN DCs (n=6), small intestinal LP APCs (n=6-10) and CD11c+MHCII- cells (n=4) within the lymphocyte gate harvested from 11c$^{AhR+/+}$ (Cre-) or 11c$^{AhR-/-}$ (Cre+) littermates as shown. Values are mean ± SEM, Mann-Whitney test: ns, not significant. FACS sorted populations from respective groups as indicated where presence or absence of floxed *Ahr* exon 2 were probed by PCR and products separated by gel electrophoresis (n=2). FACS plot showing gating strategy for lymphocytes and subsequent quantification of CD11c+MHCII- cells, where values show mean ± SEM (n ≥12). Student's t-test: ns, not significant. Mac, Macrophages.

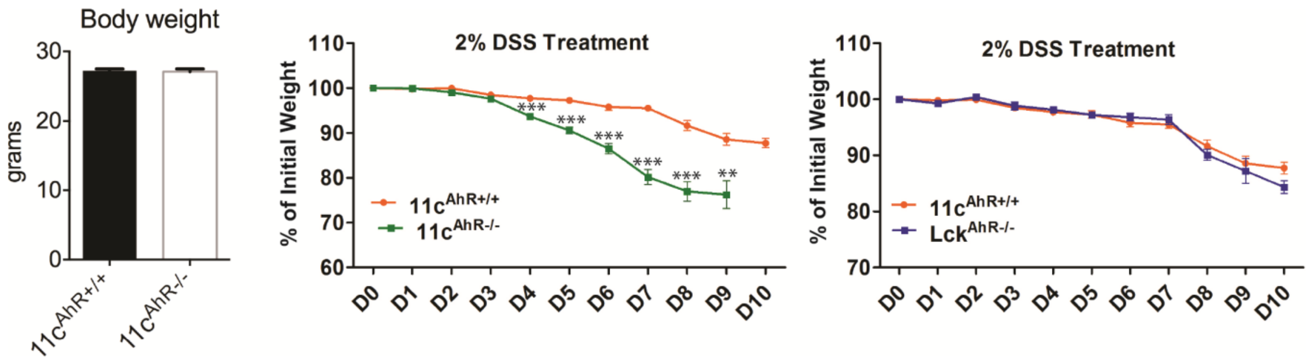

Supplementary Figure S2. 2% DSS induced colitis in mice. Body weight of males measured in grams at 10 weeks of age. Values are mean ± SEM for respective groups (n =6). Graphs depicting daily percentage of initial weight of 11c$^{AhR-/-}$ and Lck$^{AhR-/-}$ mice against the control group 11c$^{AhR+/+}$ with all statistical differences as shown where applicable (n ≥8 per group), Student's t-test: **, P < 0.01; ***, P < 0.001.

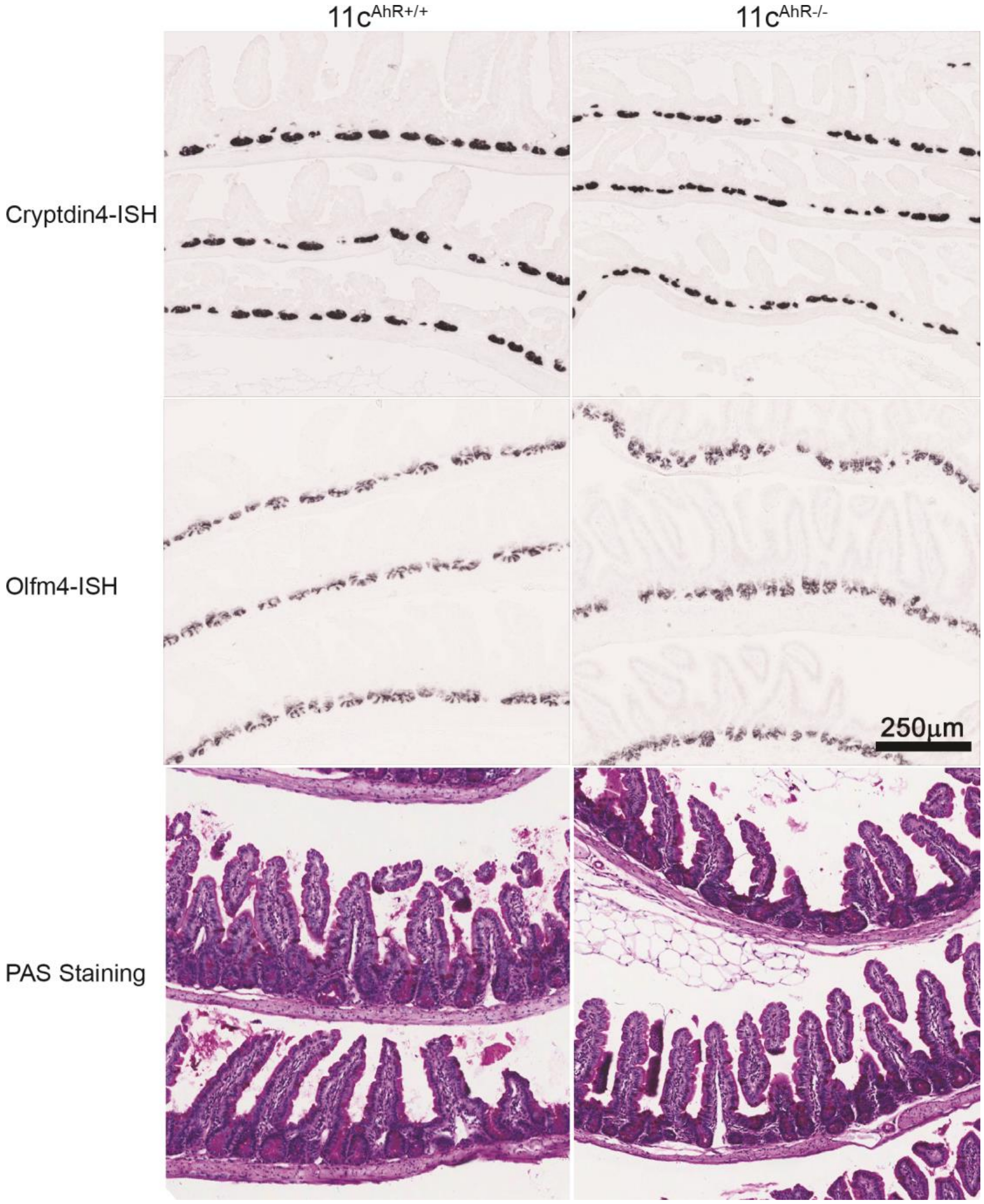

Supplementary Figure S3. Extended tracts of small intestinal sections comparing 11c$^{AhR+/+}$ mice with littermate controls. Representative Ileal sections of respective groups as shown for *in situ* hybridizations performed to detect Paneth cells (top panel), Stem Cells (middle panel) and carbohydrate-rich cells such as Goblet cells in the villus (bottom panel) via PAS staining.

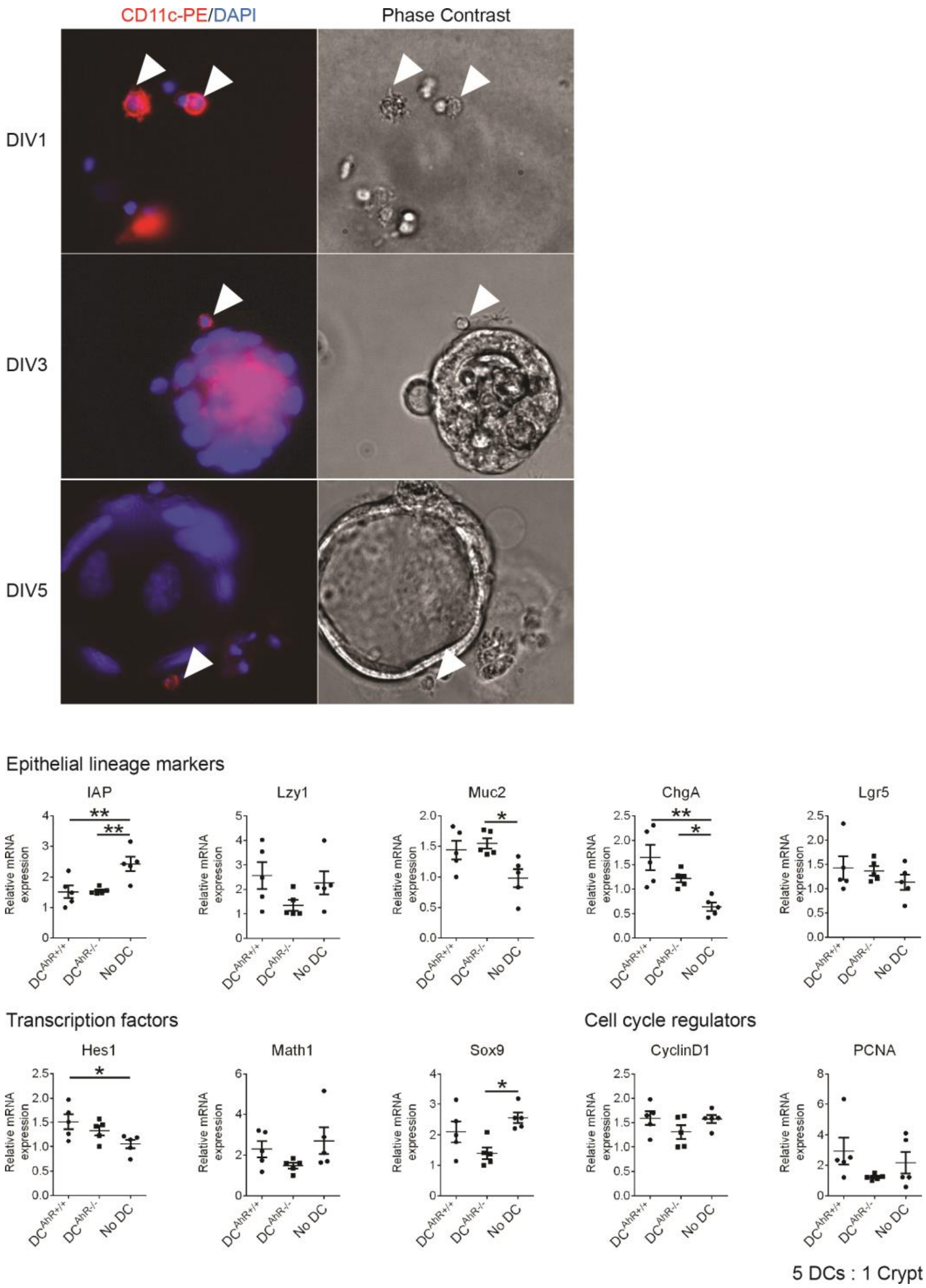

Supplementary Figure S4. Co-culture experiments reveal DC- and AhR- dependent effects on *ex vivo* organoid growth and differentiation. Immunofluorescence images of dendritic cells (red and arrows) embedded in Matrigel with co-cultured organoids on days *in vitro* (DIV) 1, 3 and 5, counterstained with DAPI to show nuclei. RT-PCR assays were performed on DIV5 samples and each data point represents a single biological replicate and presented as mean ± SEM. One-way ANOVA with Dunnett multiple comparisons tests: *, $P<0.05$; **, $P<0.01$ where 'No DC' group serves as the control for mean comparisons were performed as shown. Mann-Whitney tests for mean comparisons between $DC^{AhR+/+}$ and $DC^{AhR-/-}$ groups were not statistically significant except for PCNA ($P<0.05$).

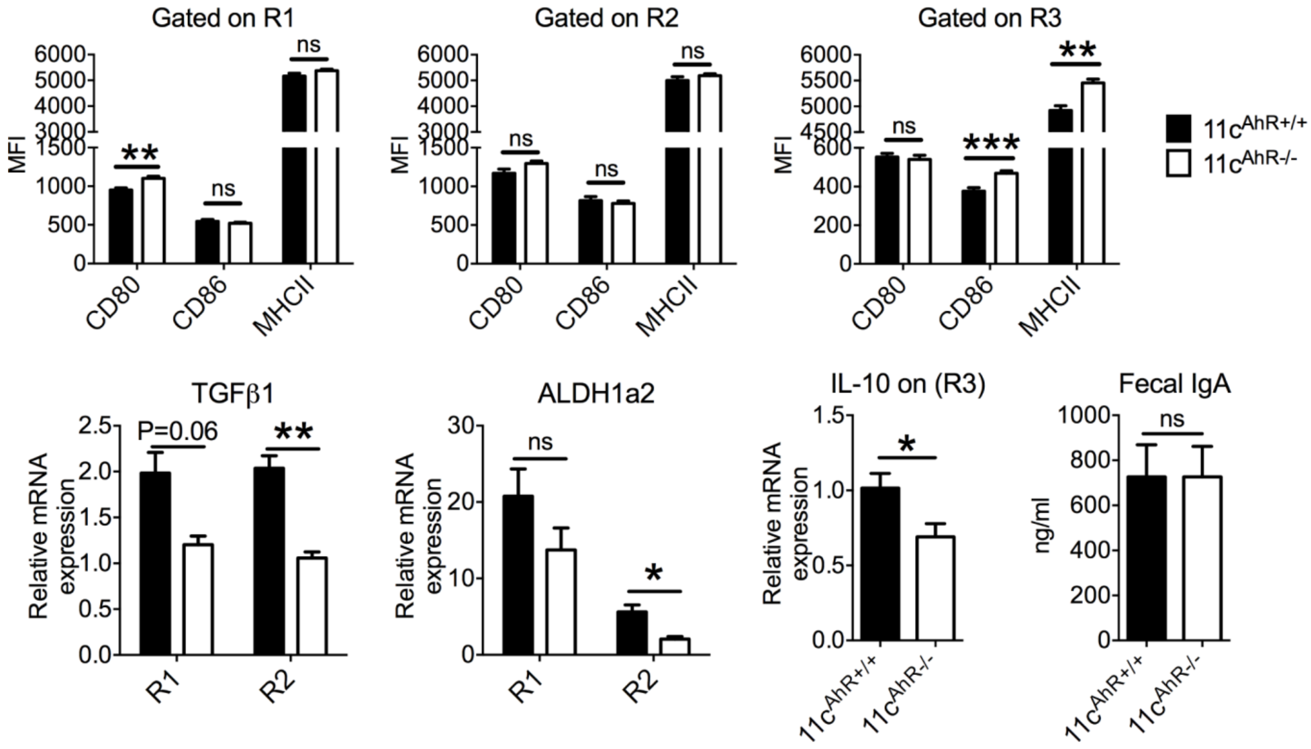

Supplementary Figure S5. AhR-deficient APCs display a more activated, immunogenic phenotype. Maturation markers expression levels shown as MFI ± SEM pooled from 3-5 independent experiments from respective gated APC subsets as shown (n =5-13), Mann-Whitney test; **, P < 0.01; ***, P < 0.001. Quantitative RT-PCR assays on *Tgfβ1*, *Aldh1a2* and *Il10* expression in FACS sorted APC subsets accordingly. Bar graphs represent mean ± SEM from 2 independent experiments (n =4-5), Mann-Whitney test: *, P < 0.05; **, P < 0.01. Mean fecal-IgA concentration ± SEM as detected by ELISA is shown. Data presented is representative of two independent experiments (n ≥10), Student's t-test: ns, not significant

**Fluidigm 48.48 array**			
Category (n)	Gene names:	Forward Primer 5’-3'	Reverse Primer 5'-3'
Wnt receptors and mediators (13)	Fzd3	TGTCCGTACCAGGTTACTCAGATGA	GAATCCCAACTATGAGAGCCATGA
	Fzd6	TGGACACTTTTGGCATCCGA	TATAGCCTTGGTCCCCGGAA
	Fzd9	TAAGGACTTCGCGCTGGTTT	AAGATAATCGGGCGCTCTGG
	Fzd10	GCATGGCCAGCTCTTTATGG	GTTGGCTTCAATGGCCTCAT
	Wnt3	CAAGCACAACAATGAAGCAGGC	TCGGGACTCACGGTGTTTCTC
	Wnt3a	TCGGAGATGGTGGTAGAGAAA	CGCAGAAGTTGGGTGAGG
	Wnt2b	CACCCGGACTGATCTTGTCT	GCCACAACACATGATTTCACA
	Wnt5b	GACGCCAACTCCTGGTGGT	GCATAACTCTGCCAAAGACAGATG
	Wnt7a	CGACTGTGGCTGCGACAAG	CTTCATGTTCTCCTCCAGGATCTTC
	Wnt8b	TTGGGACCGTTGGAATTGCC	AGTCATCACAGCCACAGTTGTC
	Wnt9a	GCAGCAAGTTTGTCAAGGAGTTCC	GCAGGAGCCAGACACACCATG
	Wnt11	CGTGTGCTATGGCATCAAGT	GCTCGATGGAGGAGCAGTTC
	Dkk3	CGAGAGGTGGAGGAGCTGATG	GTCTCCGTGCTGGTCTCATTG
Wnt Targets (6)	Axin2	GAGCAGCTCAGCAAAAAGGG	ACTGTCTCGTCGTCCCAGAT
	BMP4	CTTGAGTACCCGGAGCGTC	AAAGCAGAGCTCTCACTGGTC
	Sox9	AGGAAGCTGGCAGACCAGTA	TCCACGAAGGGTCTCTTCTC
	C-myc	CAGCCCTGAGCCCCTAGT	TCTCCACAGACACCACATCAA
	Wisp1	TGATGACGCAAGGAGACCAC	CCGGGCATTGACGTTAGAGA
	Wisp2	GTTTTGTGCCGCTGTGATG	CTGAGGAGGGCTGGATTG
Cytokines (8)	TNFα	CAAATGGCCTCCCTCTCAT	CTCCTCCACTTGGTGGTTTG
	IL-1β	GCTGAAAGCTCTCCACCTCA	GGCCACAGGTATTTTGTCGT
	IL-6	CAAAGCCAGAGTCCTTCAGA	GAGCATTGGAAATTGGGGTA
	IL-12A	GTGTCAATCACGCTACCTCC	TTTCTCTGGCCGTCTTCACC
	IL-23A	GCACCTGCTTGACTCTGACA	ATCCTCTGGCTGGAGGAGTT
	TGF-β1	GTGTGGAGCAACATGTGGAA	CGTCAAAAGACAGCCACTCA
	IL-10	ATCGATTTCTCCCCTGTGAA	TCATTCATGGCCTTGTAGACAC
	IFNγ	ATGAACGCTACACACTGCATC	CCATCCTTTTGCCAGTTCCTC
Enzymes and TFs (4)	ALDH1a2	ACCGTGTTCTCCAACGTCACTGAT	TGCATTGCGGAGGATACCATGAGA
	Blimp1	GACGGGGGTACTTCTGTTCA	GGCATTCTTGGGAACTGTGT
	IDO1	CCATGGCGTATGTGTGGAAC	AGAGCTCGCAGTAGGGAACA
	IRF4	AATCCCCATTGAGCCAAGCA	TCGTCGTGGTCAGCTCTTTC
Notch Signalling (7)	Notch1	CGGTGAACAATGTGGATGCT	ACTTTGGCAGTCTCATAGCT
	Notch2	GTGGAGGCGACTCTTCTGCT	GCTGGGAGTCACGTTATACT
	Jagged1	GCCGAGGTCCTACACTTTGCT	GTGGGCAATCCCTGTGTTTT
	Jagged2	GAGGTCAAGGTGGAAACAGT	TGTCCACCATACGCAGATAA

	Hes1	TCAGCGAGTGCATGAACGAG	CATGGCGTTGATCTGGGTCA
	Hey1	TGAGCTGAGAAGGCTGGTAC	ACCCCAAACTCCGATAGTCC
	Dll4	GCACCAACTCCTTCGTCGTC	GTTTCCTGGCGAAGTCTCTG
AhR pathway (4)	AhR	AGCCGGTGCAGAAAACAGTAA	AGGCGGTCTAACTCTGTGTTC
	Cyp1a1	CAGGATGTGTCTGGTTACTTTGAC	CTGGGCTACACAAGACTCTGTCTC
	Cyp1a2	CCATGTGCTTTGGGAAGAACTT	GTCCTTGCTGTTATTCACGATGTT
	Cyp1b1	CCGAAAAGAAAGCGTCTGG	GAACATCCGGGTATCTGGTAAA
Housekeeping genes (6)	Ppia	CACCGTGTTCTTCGACATCA	CAGTGCTCAGAGCTCGAAAGT
	Polr2α	TGGTCCTTCGAATCCGCATC	GGACTCAATGCATCGCAGGA
	Hprt	AGCCAAATACAAAGCCTAAGATGA	GGACGCAGCAACTGACAT
	Tbp	GCACAGGAGCCAAGAGTG	AGGGAACTTCACATCACAGC
	Hmbs	CCACGGGAGACAAGATTC	ACAGCATCACAAGGGTTT
	β-actin	CTGTATTCCCCTCCATCGTG	CCTCGTCACCCACATAGGAG
**Co-culture experiments**			
Epithelial lineage markers	Lzy1	GAGACCGAAGCACCGACTATG	CGGTTTTGACATTGTGTTCGC
	ChgA	CCCGAAGTGACTTTGAGGAA	GATGGCTGACAGGCTCTCTA
	Muc2	ACAAAAACCCCAGCAACAAG	GAGCAAGGGACTCTGGTCTG
	IAP	GGTATCATCCCAGCTGAAGAG	CATCCCGTCTCCCAGGAA
	Lgr5	CCCAATGCGTTTTCTACGTT	AGGCTCGGTTCCCTGTTAAT
Transcription factor	Math1	TCCCGTCCTTCAACAACGAC	CTCTCCGACATTGGGAGTCTG
Proliferation markers	PCNA	ACCTCACCAGCATGTCCAA	TCTTGATTTGGTGCTTCGAATA
	Cyclin-D1	AGTGCGTGCAGAAGGAGATT	AGGAAGCGGTCCAGGTAGTT

Supplementary Table 1
